# Prevalence and DALYs of skin diseases in Ubonratchathani based on real-world national healthcare service data

**DOI:** 10.1038/s41598-022-20237-0

**Published:** 2022-10-08

**Authors:** Nutchada Prasitpuriprecha, Sumonman Santaweesuk, Prasit Boonkert, Parinya Chamnan

**Affiliations:** 1 Department of Social Medicine, Sunprasitthiprasong Regional Hospital, Ubonratchathani, Thailand; 2 Strategy and Policy Division, National Health Security Office Region 10, Ubonratchathani, Thailand; 3 Cardiometabolic Research Group, Department of Social Medicine, Sunprasitthiprasong Regional Hospital, Ubonratchathani, 34000 Thailand; 4 College of Medicine and Public Health, Ubonratchathani University, Ubonratchathani, Thailand

**Keywords:** Diseases, Skin diseases, Public health, Epidemiology, Health care, Health care economics

## Abstract

There is little evidence to describe the burden of skin diseases in developing countries and its accuracy remained uncertain. We aimed to examine prevalence and disability adjusted life years (DALYs) of skin diseases in a Thai general population in Ubonratchathani. Based on real-world healthcare service data (diagnoses, prevalence, and cause-specific mortality) retrieved from the National Health Security Office reimbursement database, we used a simplified prevalence-based approach adopted in the Global Burden of Diseases to compute disease burden, measured as DALYs, of skin diseases. DALYs was calculated as the sum of years lost due to disability and years of life lost due to skin diseases, with adoption of previously published averaged disability weights and a 95% uncertainty interval (UI) estimated using a Bayesian bootstrap technique. From a total population of 1,503,945, 110,205 people were affected by skin disease in 2018—an overall prevalence of 7%. The prevalence varied across sex, age group and geographic areas. The most common skin diseases treated in Ubonratchathani’s healthcare services were dermatitis, bacterial skin diseases and urticaria (prevalence of 2.35%, 2.21% and 0.89% respectively). Overall DALYs of skin diseases in Ubonratchathani population was 26,125 (95%UI 24,783–27,467), and this was relatively higher in men than women. (DALYs 13,717 (12,846–14,588) and 12,408 (11,417–13,399) for men and women respectively). The greatest contributors of DALYs were cellulitis, decubitus ulcer and contact dermatitis (11,680, 4,806 and 1,598 years respectively). In conclusion, skin disease caused substantial disease burden in this Thai population, with cellulitis being the largest contributor.

## Introduction

Skin disease is an important public health problem worldwide. The disease affects people of all ages and genders from different ethnicities and countries, particularly in tropical regions^1^. Skin diseases are common and can be presented in all level of healthcare services partly due to their symptoms and signs being easily recognized. According to the 2010 Global Burden of Disease (GBD) report, three skin diseases were among the top 10 most prevalent diseases globally^2^. Albeit generally low mortality of skin diseases, many skin disorders are chronic conditions and may be associated with reduced quality of life^3^ and substantial morbidity^3,4^. In effect, skin diseases could lead to significant years lost due to disability (YLD)—they were among the leading causes of YLDs worldwide^5^.

According to GBD of Skin Diseases 2010, skin diseases contributed to substantial burden with YLD of more than 33.7 million years, the fourth among all diseases worldwide. Skin conditions collectively resulted in the disability adjusted life years (DALYs) of 36.9 million years, being the 18th leading cause of global disease burden. The GBD 2017 Report showed that skin and subcutaneous diseases caused 44.1 million years of DALYs. The GBD of Skin Diseases 2010 and GBD 2017 Reports described indices of disease burden in different global regions and both developed and developing countries^2,6^. A number of previous studies examining temporal changes in disease burden suggested that the number of DALYs for skin diseases was not improved or even increased, with varying burdens across types of skin diseases and sexes. This was similar for both developed and developing countries^4^. According to Thailand’s 2014 Burden of Diseases Report^7^, the top three leading causes of YLDs were alcohol dependence and harmful use, osteoarthritis and diabetes for Thai men, while osteoarthritis, diabetes and dementia for Thai women. Patterns of years of life lost due to premature mortality (YLLs) and DALYs in a Thai population were similar, with the top three causes being road traffic accidents, stroke and HIV/AIDS for men, and stroke, diabetes and ischemic heart disease for women. Interestingly, skin disease was not in the top 20 contributors of any of these measures of disease burdens in both sexes, and there is a lack of evidence on the comparative burden of different types of skin disease in the Thai population.

 Methods and parameters used to inform computations of DALYs in the previous studies, although being consecutively updated and improved, were largely based on expert panels, epidemiological studies, specific analytical methods and disability weighting techniques, which may be somewhat problematic^5,8^. This would particularly have considerable impact on DALYs estimates in developing countries, where the quality and completeness of data sources (prevalence, incidence, disability and cause-specific mortality) may be unsatisfactorily low. This merits further efforts to improve these country-specific data to obtain more accurate and reliable estimates of disease burden. This study aimed to examine the prevalence and DALYs of skin diseases in a Thai general population using real-world healthcare service data. This would help inform policy decision and planning to address skin and subcutaneous diseases in tropical and developing countries.

## Methods

This study examined the burden of skin diseases in a Thai general population in Ubonratchathani using data from health services and reimbursement database. Ubonratchathani locates in the Northeastern region of Thailand and is the country’s fourth largest province with a total population of 1,503,945 in 2018. Health service data have been standardized and stored in electronic health records in all levels of healthcare services, primary to tertiary care, for more than 15 years. Service data include personal and medical history, physical examination, investigations, diagnoses, medical treatments and procedures, follow-up, health promotion and disease prevention activities and vital status. All the service data are entered and stored in 43 separated folders, each of which is linked with unique national identification numbers. The data are then retrieved through a centralized database system called ‘Health Data Center’ and assembled and audited in a database of the National Health Security Office (NHSO) for reimbursement purpose.

All methods in this study were performed in compliance with the International Conference on Harmonization (ICH) and the principles of the Declaration of Helsinki. After the approval from the Sunprasitthiprasong Hospital Ethics Committee (Ref no. 043/2557), demographic data, skin disease diagnoses and vital status for 1,503,945 residents of Ubonratchathani were anonymously retrieved from the NHSO database with assistance from the NHSO’s IT manager. Diagnostic data included whether an individual was diagnosed with skin diseases, diagnoses of skin diseases according to the 10th revision codes of the International Classification of Diseases (ICD-10 codes) and respective visit dates between 1st January 2018 and 31st December 2018. Vital status (dead/alive, causes and date of death) was obtained and cross-validated with national death certificates. In 2006, the Public Health Office of Ubonratchathani in collaboration with the University of Queensland started a project called ‘SPICE-COD’, using a verbal autopsy technique by local health care officers to improve the accuracy of causes of deaths data in Ubonratchathani. A validation study by Muangmai W, et al. suggested that the accuracy of data on causes of death significantly improved from 59% in 2007 to 71% in 2009, with a substantial reduction in the proportion of unknown causes of death^9^. This approach has been put in place since 2006 and it has helped assure accurate and reliable data on vital status and causes of death in Ubonratchathani’s population.

Sample size determination was based on research questions to examine the prevalence of different skin diseases according to the GBD 2017. Based on data on prevalence of 18 skin diseases reported in a study by Fasih et al.^10^ with a 95% confidence and 10% margin of error allowed, a sample size ranging from 1509 to 63,641 would be needed for this study^11^. However, we decided to use data on all individuals residing in Ubonratchathani in 2018 as this would increase the study power and reduce biased selection at no additional expenses.

### Classification of skin diseases

Based on the GBD 2017, 22 skin and subcutaneous diseases were selected and classified according to the ICD-10 codes obtained from the NHSO database (Supplementary Table 1). These included dermatitis (atopic dermatitis, seborrheic dermatitis, contact dermatitis), viral skin diseases (wart, molluscum contagiosum), fungal skin diseases (tinea capitis, other fungal skin diseases), bacterial skin diseases (impetigo, pyoderma, cellulitis, abscess and other bacterial skin diseases), acne vulgaris, psoriasis, alopecia areata, scabies, melanoma, squamous cell carcinoma and basal cell carcinoma, carcinoma in situ, pruritus, urticaria, decubitus ulcer and other skin and subcutaneous diseases. According to current ICD coding systems, squamous cell carcinoma and basal cell carcinoma are not coded as separate diagnoses as the two malignancies are captured under a single diagnostic code of non-melanoma skin cancers.

### Estimation of prevalence of skin diseases and disease-specific mortality

Based on the NHSO database, the overall and cause-specific prevalence of skin diseases was calculated as the number of individuals diagnosed with skin diseases divided by a total population of Ubonratchathani, with 95% uncertainty interval (UI) estimated using the Bayesian bootstrap technique (normal distribution assumed). The prevalence estimates were computed for the overall population and by sexes and districts. According to Karimkhani et al.^12^ age- and sex-specific mortality was obtained for six skin and subcutaneous diseases: cellulitis, decubitus ulcer, abscess and other bacterial skin diseases, melanoma, squamous cell carcinoma, and other skin and subcutaneous diseases. This was based on mortality observed in individuals diagnosed with the above skin diseases. Those with each of these diagnoses in 2018 were tracked if they were dead or alive in the following years after diagnosis. Data on vital status (dead or alive) and date of death as well as causes of death, if a person died, were obtained for each individual. A previous validation study under the Setting Priority using Information on Cost-Effectiveness (SPICE) project showed that data on causes of deaths in Ubonratchathani were satisfactorily accurate^9^.

### Estimation of DALYs attributable to skin diseases

Using a simplified approach to calculation of DALYs (prevalence-based) adopted in the GBD 2010^13^, we computed DALYs as the sum of years lost due to disability (YLDs) and years of life lost (YLLs) due to skin diseases for each sex, age group, and type of skin diseases. The prevalence estimates were multiplied by averaged disability weights to calculate YLDs resulting from each type of skin disease. The averaged disability weights were derived from the GBD 2017^14^. Due to disease severity not being captured in electronic health records for most skin diseases, a single constant value of averaged disability weight was applied for each individual skin disease. YLLs were calculated as the sum of each death multiplied by the standard life expectancy at each age. As described earlier, deaths due to the selected skin diseases were identified through the NHSO database. The standard life expectancy for each sex and 5-year age group was taken from the Public Health Statistic A.D. 2018 Report convened by Thailand’s Ministry of Public Health (Strategy and Planning Division)^15^. Corresponding 95% UI for these measures of disease burden were calculated using a parametric bootstrap technique^16^. All analyses were performed using STATA 14.2 statistical package.

## Results

### Prevalence of skin diseases

Out of 1,503,945 people residing in Ubonratchathani, 110,205 were diagnosed with at least one of skin diseases, a prevalence of 7.33% (95%UI 7.29–7.37). Table 1 shows the number and prevalence of skin diseases diagnosed and treated in Ubonratchathani’s healthcare services in 2018. The prevalence of skin diseases varied substantially from lower than 0.01% to 2.35%. The highest prevalence was observed for dermatitis, bacterial skin infection and urticaria (2.35%, 2.21% and 0.89% respectively). Contact dermatitis constituted most cases of all dermatitis, with the prevalence of 0.98%, Considering skin malignancies, 435 cases of malignant melanoma were treated in Ubonratchathani’s healthcare services including a special cancer hospital, accounting for a prevalence of 0.03% or 30 per 100,000 population. There were 222 cases of non-melanoma skin cancers (squamous and basal cell carcinoma combined), a prevalence of 10 per 100,000 population.

**Table 1:** Numbers and prevalences of 15 skin diseases diagnosed and treated in Ubonratchathani’s healthcare services in 2018.

Skin diseases	Number of cases	Prevalence (95% UI)^a^
**Total cases of skin diseases**	110,205	7.33 (7.29–7.37)
**1. Dermatitis**	35,411	2.35 (2.33–2.38)
Atopic dermatitis	969	0.06 (0.06–0.07)
Seborrheic dermatitis	607	4.04 (3.73–4.37) × 10^–2^
Contact dermatitis	14,795	0.98 (0.97–1.00)
**2. Viral skin diseases**	9466	0.63 (0.62–0.64)
Wart	496	0.03 (0.03–0.04)
Molluscum contagiosum	12	7.98 (4.53–14.0) × 10^–4^
**3. Fungal skin diseases**	13,360	0.89 (0.87–0.90)
Tinea capitis	373	0.02 (0.02–0.03)
Other fungal skin infection	13,047	0.87 (0.85–0.88)
**4. Bacterial skin diseases**	33,294	2.21 (2.19–2.24)
Cellulitis	15,641	1.04 (1.02–1.06)
Pyoderma	13,958	0.93 (0.91–0.94)
Impetigo	5680	0.38 (0.37–0.39)
Abscess and other bacterial skin diseases	14	9.31 (5.51–15.7) × 10^–4^
**5. Melanoma**	435	2.89 (2.63–3.18) × 10^–2^
**6. Non-melanoma skin cancers**^b^	222	1.48 (1.29–1.68) × 10^–2^
**7. Carcinoma in situ**	21	1.40 (0.91–2.14) × 10^–3^
**8. Other skin and subcutaneous disease**	15,502	1.03 (1.01–1.05)
**9. Pruritus**	5244	0.35 (0.34–0.36)
**10. Urticaria**	13,380	0.89 (0.87–0.90)
**11. Decubitus ulcer**	577	3.84 (3.54–4.16) × 10^–2^
**12. Acne vulgaris**	92	7.83 (7.40–8.29) × 10^–2^
**13. Psoriasis**	1178	0.08 (0.07–0.08)
**14. Alopecia areata**	176	1.17 (1.01–1.36) × 10^–2^
**15. Scabies**	195	1.30 (1.13–1.49) × 10^–2^

^a^Prevalence is presented in percent and 95%UI calculated using Bayesian bootstrap technique.

^b^Non-melanoma skin cancers; squamous cell carcinoma and basal cell carcinoma.

**Figure 1:**
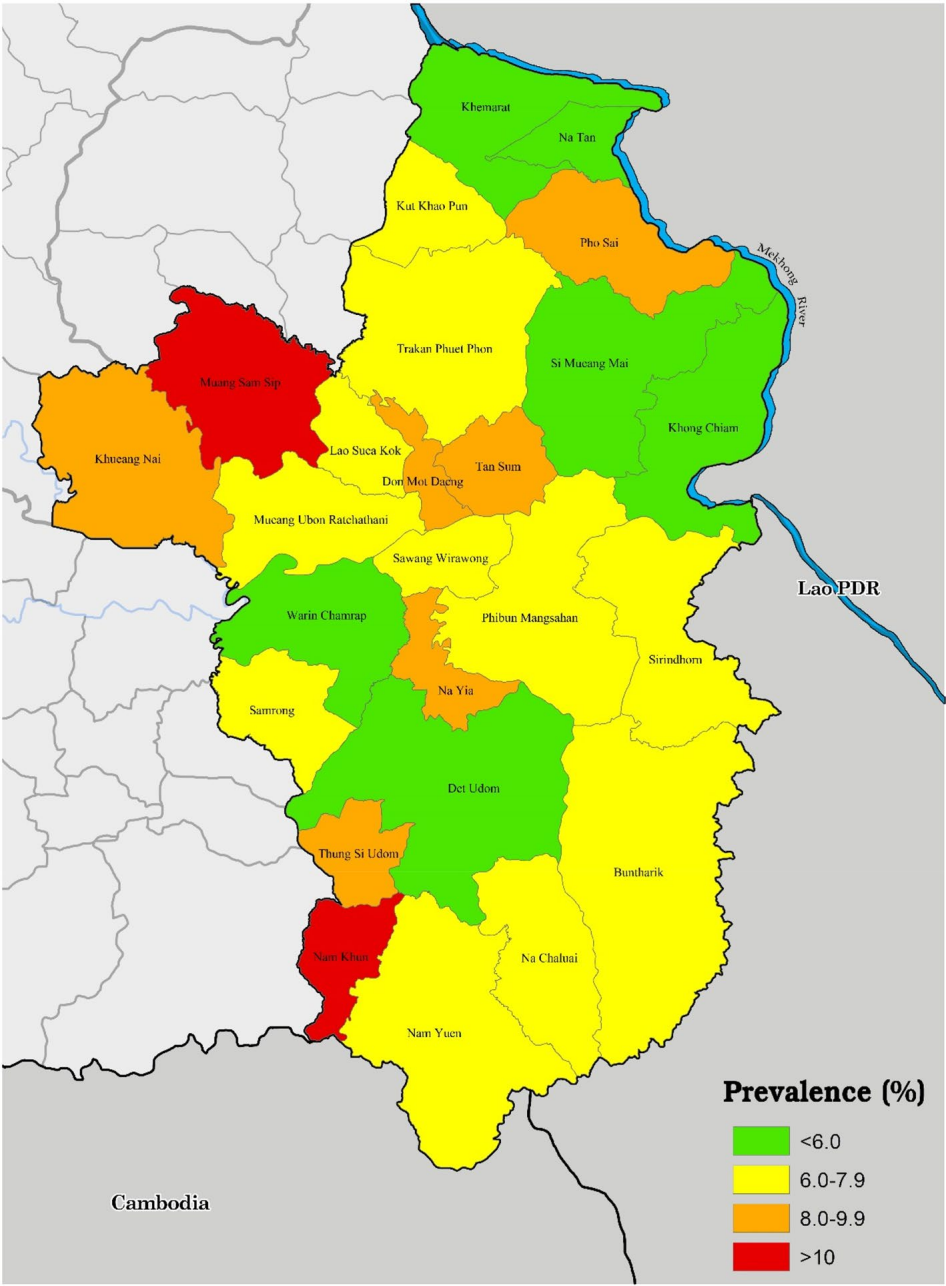
Prevalence of skin diseases across different districts of Ubonratchathani. This map was generated using QGIS version 3.22.10.

Figure 1 shows the prevalence of skin diseases by district. There was great discrepancy in the prevalence of skin diseases across 25 districts of Ubonratchathani, ranging from 5.35 to 15.85%. The districts close to the border between Thailand and Lao PDR appeared to have lower skin disease prevalence than those located further from the border.

### YLLs, YLD and DALYs attributable to skin diseases

Numbers of deaths and YLLs for six skin diseases overall and by sex are shown in Supplementary Table 2. Most deaths and YLLs were from cellulitis—606 deaths contributing to 10,695 years of life lost, Decubitus ulcer and other skin and subcutaneous diseases contributed to 4654 and 4535 years of life lost. With a relatively small number of deaths from squamous/basal cell carcinoma and melanoma (35 and 24 deaths), 590 and 393 years of life were lost due to the two skin malignancies, while there were no deaths from abscess and other bacterial skin diseases. Males contributed to more YLLs than females similarly for all the diseases. Years lost due to disability from skin diseases in Ubonratchathani was as high as 5259 (95%UI 5206—5312) years (Supplementary Table 3). The top 3 diseases that contributed most YLDs were contact dermatitis, urticaria and cellulitis (YLDs 1598, 1445 and 985 years). These together contributed to 65% of all YLDs. Melanoma resulted in a total YLD of 144 years. When using a disability weight of squamous cell carcinoma, non-melanoma skin cancers contributed to 48 YLDs. In a sensitivity analysis using a disability weight of basal cell carcinoma, the diseases caused only 2.4 YLDs. Of note, females had higher YLDs than males for all skin diseases, except for cellulitis, impetigo, psoriasis and decubitus ulcer.

**Figure 2:**
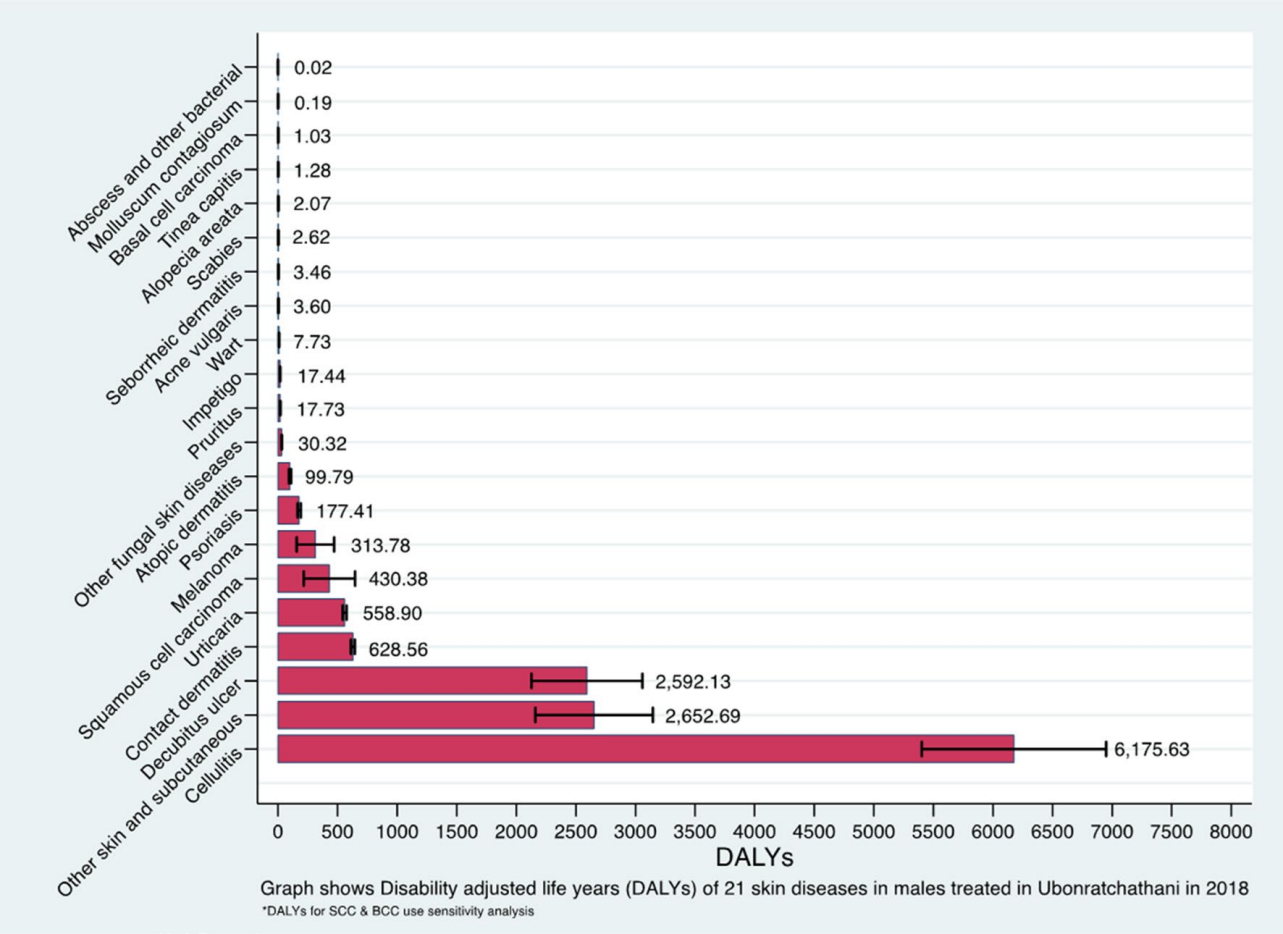
DALYs due to different skin diseases in males (in ascending order).

**Figure 3:**
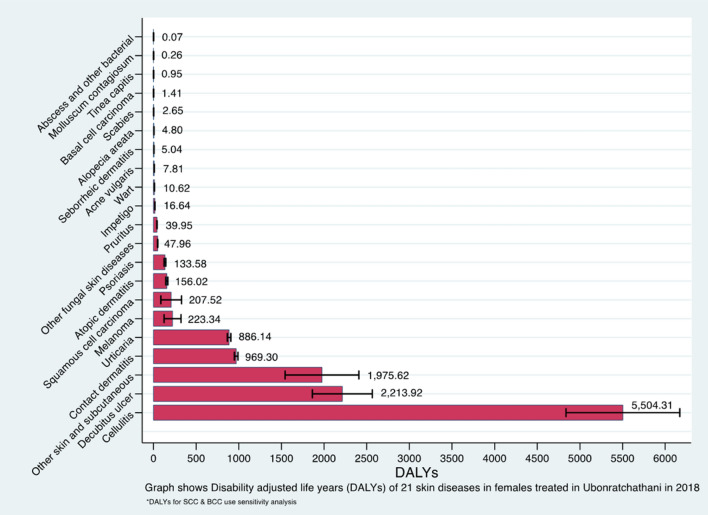
DALYs due to different skin diseases in females (in ascending order).

Table 2 shows the estimated YLLs, YLDs and DALYs due to skin diseases in the population of Ubonratchathani, overall and by sex. The all-age all-cause DALYs in 2018 was 26,124.7 (95%UI 24,782.8—27,466.6). Overall, males had a slightly higher level of DALYs than females (DALYs 13,716.8 (12,845.6–14,587.9) and 12,407.9 (11,416.7–13,399.1) respectively) The largest contribution of s single cause to DALYs in Ubonratchathani province was from cellulitis, which accounted for up to 45% of total DALYs. This was followed by decubitus ulcer and contact dermatitis, which together contributed to another 25% of the total DALYs. For certain skin diseases including cellulitis, decubitus ulcer and skin cancers, they contributed to more DALYs in males than females, while other diseases caused more DALYs in females than males. Figures 2 and 3 show DALYs due to different skin diseases in ascending order in males and females. Considering a single cause, cellulitis, decubitus ulcer and contact dermatitis similarly caused most DALYs in both males and females, with the top three diseases accounted for almost 70% of the total DALYs in each sex.

**Table 2:** YLL, YLD and DALYs of 21 skin diseases in Ubonratchathani, overall and by sex.

Skin diseases	Females			Males			Total DALYs (95% UI)
	YLL (95% UI)	YLD (95% UI)	DALYs (95% UI)	YLL (95% UI)	YLD (95% UI)	DALYs (95% UI)	
Atopic dermatitis	NA	156.02 (142.71–169.34)	156.02 (143.74–168.31)	NA	99.79 (89.59–110.00)	99.79 (90.63–108.95)	255.82 (241.06–270.57)
Contact dermatitis	NA	969.30 (947.81–990.79)	969.30 (948.37–990.23)	NA	628.56 (611.06–646.06)	628.56 (612.10–645.02)	1597.86 (1571.46–1624.26)
Seborrheic dermatitis	NA	5.04 (4.47–5.61)	5.04 (4.51–5.57)	NA	3.46 (2.94–3.98)	3.46 (3.06–3.86)	8.50 (7.84–9.15)
Psoriasis	NA	133.58 (122.30–144.87)	133.58 (123.37–143.80)	NA	177.41 (165.00–189.82)	177.41 (164.38–190.44)	310.99 (293.47–328.52)
Cellulitis	5030.74 (4671.70–5389.78)	473.57 (462.48–484.67)	5504.31 (4836.89–6171.73)	5663.82 (5336.04–5991.60)	511.81 (500.01–523.61)	6175.63 (5402.46–6948.80)	11,679.94 (10,855.02–12,504.86)
Impetigo	NA	16.64 (15.98–17.30)	16.64 (15.94–17.34)	NA	17.44 (16.79–18.08)	17.44 (16.81–18.06)	34.08 (33.25–34.91)
Abscess and other bacterial skin disease	NA	0.07 (0.03–0.10)	0.07 (0.02–0.11)	NA	0.02 (0.00–0.04)	0.02 (0.00–0.03)	0.08 (0.04–0.13)
Scabies	NA	2.65 (2.02–3.27)	2.65 (2.20–3.09)	NA	2.62 (2.02–3.22)	2.62 (2.09–3.15)	5.27 (4.56–5.97)
Tinea capitis	NA	0.95 (0.80–1.10)	0.95 (0.80–1.11)	NA	1.28 (1.07–1.50)	1.28 (1.10–1.47)	2.24 (2.05–2.43)
Other fungal skin disease	NA	47.96 (46.95–48.97)	47.96 (46.78–49.14)	NA	30.32 (29.60–31.05)	30.32 (29.52–31.13)	78.28 (76.93–79.63)
Wart	NA	10.62 (9.30–11.94)	10.62 (9.37–11.87)	NA	7.73 (6.81–8.65)	7.73 (6.67–8.79)	18.35 (16.78–19.93)
Molluscum contagiosum	NA	0.26 (0.05–0.47)	0.26 (0.08–0.44)	NA	0.19 (0.04–0.33)	0.19 (0.01–0.36)	0.44 (0.17–0.72)
Acne vulgaris	NA	7.81 (5.99–9.64)	7.81 (6.27–9.36)	NA	3.60 (2.40–4.79)	3.60 (2.19–5.00)	11.41 (8.73–14.08)
Alopecia areata	NA	4.80 (3.77–5.82)	4.80 (3.78–5.81)	NA	2.07 (1.50–2.63)	2.07 (1.65–2.49)	6.86 (5.93–7.80)
Pruritus	NA	39.95 (38.81–41.10)	39.95 (38.43–41.47)	NA	17.73 (16.82–18.65)	17.73 (16.82–18.64)	57.68 (56.45–58.92)
Urticaria	NA	886.14 (869.41–902.87)	886.14 (866.41–905.87)	NA	558.90 (543.36– 574.44)	558.9 (542.05–575.75)	1445.04 (1179.77– 1710.32)
Decubitus ulcer	2141.85 (1964.77–2318.94)	72.07 (63.37–80.78)	2213.92 (1862.04– 2565.80)	2511.87 (2272.39–2751.35)	80.26 (72.85–87.66)	2592.13 (2126.77– 3057.48)	4806.05 (4093.29– 5518.81)
Other skin and subcutaneous disease	1921.82 (1719.76–2123.88)	53.80 (52.41–55.19)	1975.62 (1543.82– 2407.42)	2613.48 (2329.76–2897.20)	39.21 (38.22–40.20)	2652.69 (2159.04– 3146.34)	4628.312 (4004.29– 5252.34)
Non-melanoma skin cancers^a^	179.62 (127.89–231.35)	27.90 (22.58–33.22)	207.52 (85.75–329.30)	409.89 (310.63–509.15)	20.49 (16.79–24.20)	430.38 (214.67–646.10)	637.91 (438.49–837.32)
Melanoma	120.42 (91.99–148.85)	102.92 (90.89–114.95)	223.34 (124.34– 322.34)	272.28 (219.97–324.59)	41.50 (34.83–48.17)	313.78 (156.66–470.90)	537.12 (362.37–711.87)
**Total**	9394.45 (8534.88– 10,254.02)	3013.472 (2978.44–3048.50)	12407.92 (11416.70–13399.14)	11,471.34 (10,155.24– 12,787.44)	2245.416 (2209.76– 2281.07)	13,716.76 (12,845.59– 14,587.92)	26,124.68 (24,782.76– 27,466.59)

95% uncertainly intervals of YLDs, YLLs and DALYs computed using a parametric bootstrap technique.

*YLDs* years lost due to disability, *YLLs* years of life lost, *DALYs*  disability adjusted life years, *NA*  not applicable.

^a^Non-melanoma skin cancers included squamous cell carcinoma and basal cell carcinoma.

The number of DALYs due to melanoma was 537.1 (362.4–711.9), with a 1.4-time higher DALYs observed in males than females. Based on the scenario using disability weight of basal cell carcinoma, basal and squamous cell carcinomas caused 637.9 (438.5–837.3) of DALYs. In a sensitivity analysis using disability weight of squamous cell carcinoma, the two diseases caused only 2.4 (2.1–2.8) of DALYs.

## Discussion

In the present study, we described the burden of skin and subcutaneous diseases in a Thai population in Ubonratchathani, using real-world healthcare service data. The overall prevalence of skin diseases was as high as 7% and varied across sex, age group and geographic areas. Dermatitis, bacterial skin infection and urticaria were the most common skin diseases. DALYs attributable to skin diseases was 26,125, and generally higher in males than females. Cellulitis, decubitus ulcer and contact dermatitis were the main causes of DALYs in this population.

Skin diseases were common worldwide, although the overall prevalence varied greatly across different countries. The overall prevalence of skin diseases in the present study was approximately 7%. This was considerably lower than the prevalence reported in other population-based survey studies, the overall prevalence of 15% and 27% reported in Bangladesh and European countries respectively^17,18^. The discrepancy may be explained by that the Bangladesh study was conducted in one rural community without systemic sampling of participants and therefore prone to biased selection—those with symptoms and signs of skin diseases were likely participating, hence high prevalence. Additionally, a population-based survey in European countries described a lifetime prevalence, whereas our study reported the prevalence of any skin diseases diagnosed and recorded in electronic health records in 2018. Differences in study settings, computation methods and definitions may make it difficult to compare skin disease prevalence across studies.

Comparison in skin disease prevalence is even more challenging when considering pattern or types of skin diseases. Based on the GBD of skin diseases 2010, fungal skin diseases, acne vulgaris and other skin and subcutaneous diseases were among the top 10 most prevalent diseases globally^2^. Dermatitis was one of the most prevalent skin diseases worldwide and the prevalence of dermatitis and its subtypes varied across populations. Dermatitis constituted 21–32% of all skin diseases^10,17,19,20^. While atopic dermatitis represented most cases of dermatitis in developed countries^6,18,21–23^, contact dermatitis constituted most cases of dermatitis in our study and some European countries^18^. Skin infestation from scabies was prevalent in developing countries^10,17,19^, but rarely occurred in developed countries. For example, the prevalence of scabies was as high as 15% in Pakistan population^10^, while the figure was lower than 1% in this Thai population and most developed countries^2^. Interestingly, warts and acne topped the ranking of the most prevalent skin diseases in European countries^18^, while they were less prevalent in developing countries^2^. These differences may suggest true discrepancies in the pattern of skin diseases between countries and geographic regions or simply reflect different age groups of study participants and methods to obtain the prevalence estimates.

Skin diseases contribute to substantial disease burden and this reportedly varied by type of skin diseases and differed across global regions and countries. Consistent with the GBD 2017 and other previous population-specific studies, our study showed that dermatitis was among major causes of DALYs, albeit different burdens being caused by its different subtypes. For example, atopic dermatitis predominated the burden of all dermatitis in developed countries^24^. while contact dermatitis topped the list of dermatitis in our study. While cellulitis contributed to 45% of DALYs due to skin diseases in the present study, it resulted in only 1% of the total DALYs from skin diseases globally^6^. This discrepancy may reflect the contribution to DALYs of high prevalence of contact dermatitis and high premature mortality from cellulitis in a Thai population in relation to other populations. This relatively high burden of cellulitis and subcutaneous bacterial infection in Thailand might simply reflect lifestyle of people in the region where traditional farming remained predominant with disproportionately high use of agricultural chemicals. This may also suggest possible differences in healthcare service systems and quality of healthcare between countries. Besides, this may be explained by differences between these studies in the quality and completeness of data on diagnoses, related consequences and cause-specific mortality used to calculate DALYs. Our findings on the top 5 causes of YLLs, YLDs and DALYs could be used to inform policy decision on resource allocation to focus more on prevention of diseases that were main causes of YLDs, such as dermatitis, and on prevention and reduction of complications and mortality related to main causes of YLLs, such as cellulitis and decubitus ulcers. This informed policy decision would be very crucial in order to effectively lower DALYs in resource-limited countries like Thailand.

Sex differences in DALYs due to skin diseases exist in many populations. This may be attributable to differences between sexes in the pattern of skin disease burden. Consistent to previous studies^2,6^, our study found that YLLs constituted higher relative contribution to DALYs in men than women. Men may be more likely to be affected by fatal skin diseases such as skin cancers, cellulitis, and other serious skin infections, while women may be more likely to suffer from non-fatal disabling skin conditions such as dermatitis, pruritus and urticaria. Interestingly, while the GBD 2017 and GBD of Skin Diseases 2010 Reports suggested that woman had higher overall burden from skin and subcutaneous diseases than men^2^, our study showed opposite results. This might reflect true differences in the patterns of disease burden caused by skin disorders in the Thai population and other populations, or might be explained by a difference in data sources and methods to compute these indices of disease burden.

Skin cancers contribute to significant burden of skin diseases, albeit with disproportionate contributions of different skin cancers in different countries. While non-melanoma skin cancers (both basal and squamous cell carcinoma) were reportedly the most common skin malignancies in many Caucasian and Asian populations^25–27^, our study showed different results with malignant melanoma predominated in this Thai population. When considering DALYs, our study is consistent with many previous studies suggesting that non-melanoma skin cancers caused a higher disease burden than malignant melanoma. According to the Burden of Cancer in Australia 2011 Report^28^, disease burden measured as DALYs of skin cancers in Australia was substantial, with melanoma being ranked number 5 and 9 cause of DALYs among cancers of all sites in males and females respectively. However, this report does not describe DALYs of melanoma and other skin cancers in relation to that of other skin diseases. While skin cancers were the top 6 and 7 contributors of DALYs from skin diseases for both males and females in this Thai population, they were not in the top 10 causes of DALYs for either sex in Mexico^29^. The discrepancy in the burden of skin cancers may be explained by the differences between countries in climates and sun exposure and protection behaviors or that the Thais may have skin type that protects them from Ultraviolet B radiation than Caucasians and other Asians^26^. It could be due to disproportionate rises in skin cancers incidence in light-skinned populations^24,30^, and also the difference across countries in healthcare system and the quality of healthcare which could impact mortality of patients with skin cancers, hence YLLs.

Consistent to the methods adopted by the 2020 WHO report on Global health estimates^31^, the prevalence-based approach to computing DALYs does not require data on disease duration and is not significantly influenced by the method to apply the discount rate; however, this approach may lead to confusion as different methods to compute YLDs and YLLs are used and YLDs can be underestimated for diseases with short duration^32^. In contrast, in the incidence-based approach YLDs and YLLs are measured consistently, but data on disease duration are essentially required for computation and incorporation of morbidities is relatively challenging. While the incidence-based approach is more useful to inform policies or interventions focusing on disease prevention, we used the simpler prevalence-based approach as it provides information on the impact of particular diseases on economic productivity at a given time.

This study was among the first to examine prevalence and burden of skin diseases measured as DALYs in people of all ages in developing countries, using the real-world healthcare service and mortality data and standard computation techniques. However, our study had some limitations. First, diagnoses of skin and subcutaneous diseases based on respective ICD-10 codes from electronic health records did not contain data on disease severity. This could have altered the estimation of YLDs and hence DALYs in our study. However, this may at least represent the burden in real-world settings. Our study used mortality derived from the NHSO data as it likely reflected age- and sex-specific all-cause mortality in this contemporary Thai population. It is possible that this may not truly represent cause-specific mortality of skin diseases of interest. This may also have altered YLLs and DALYs estimates in our study as compared to that computed using the GBD estimates. Besides, the current ICD-10 coding system did not allow separate ICD codes for squamous and basal cell carcinomas. Applying the averaged disability weight of squamous cell carcinoma to the computation of YLDs and DALYs for patients with either squamous or basal cell carcinomas would lead to underestimation of these measures of disease burden. However, a sensitivity analysis using the averaged disability weight of basal cell carcinoma would reflect the possible upper-most estimates of the disease burden of both diseases. In addition, diagnoses of skin diseases were made by various health professions from primary care nurse and medical practitioners to tertiary care medical specialists and dermatologists. Therefore, there may be uncertainty about the accuracy and completeness of diagnostic information. Of particular note, the number of cases diagnoses with acne vulgaris and molluscum was very small and may likely be underestimated. This could be because most cases of these diagnoses, particularly acne vulgaris, are not reimbursed by all types of national health insurance as they are considered aesthetic care. Hence, most patients are seeking care from over-the-counter treatment and private aesthetic and cosmetic clinics, which have been and are projected to be one of the fastest growing industries in Thailand^33^. As data used in our analyses were not include over-the-counter and private healthcare services, this could likely impact on our YLD estimates. However, all hospital diagnoses were routinely checked and coded by trained coder and then validated by the NHSO. Therefore, this method of cases ascertainment was likely to accurately capture diagnoses and burden of skin diseases in real-world clinical practices. A verification study to examine the accuracy of skin disease diagnoses may also be needed. Besides, it is important to acknowledge that the DALYs estimates based on the prevalence-based approach in our study may differ from that of the incidence-based approach. Lastly, as our study was based on data from mixed urban–rural communities in Thailand, the generalizability of our findings to other populations and countries may be limited.

## Conclusions

Skin disease caused considerable disease burden, measured as both prevalence and DALYs, in this Thai population and its contribution to the disease burden varied across sexes, age group and types of skin diseases. These estimates of disease burden will be useful in policy development and decision, health system planning and resource allocation with regards to skin diseases in Thailand and other developing countries.

## Supplementary Information

Supplementary Tables.

## Data Availability

The datasets generated and/or analysed during the current study are not publicly available due privacy or ethical restrictions but are available from the corresponding author on reasonable request.
