# Supplementary material for: Prevalence and DALYs of skin diseases in Ubonratchathani based on real-world national healthcare service data
Source: Sci Rep. 2022 Oct 8;12:16931. doi: 10.1038/s41598-022-20237-0 (PMC9547855; doi:10.1038/s41598-022-20237-0)
Supplement: Supplementary file 1 — Supplementary Tables. [file 41598_2022_20237_MOESM1_ESM.docx]

**Supplementary Table 1.** Skin and subcutaneous diseases classified with the ICD-10 code

| **Skin diseases** | **Code ICD-10** |
| --- | --- |
| **Dermatitis** | L20, L20.0, L20.8, L20.9, L21, L21.0, L21.1, L21.8, L21.9, L22, L23, L23.0 – L23.8, L23.9, L24, L24.0 – L24.8, L24.9, L25, L25.0 – L25.5, L25.8, L25.9, L26, L27, L27.0 – L27.2, L27.8, L27.9, L28, L28.0 – L28.2, L30, L30.0 – L30.5, L30.8, L30.9, L85.3, I87.2, L71.0, L98.1 |
| **Atopic dermatitis** | L20, L20.0, L20.8 |
| **Seborrheic dermatitis** | L21, L21.0, L21.1, L21.8, L21.9 |
| **Contact dermatitis** | L23, L23.0 – L23.8, L23.9, L24, L24.0 – L24.8, L24.9, L25, L25.0 – L25.5, L25.8, L25.9 |
| **Viral skin disease** | B00, B00.0 – B00.2, B00.9, B01.9, B02.9, B03, B04, B05, B05.9, B06, B06.9, B07, B08.0, B08.1, B08.2, B08.3 B08.8, B09 |
| **Wart** | B07 |
| **Molluscum contagiosum** | B08.1 |
| **Fungal skin disease** | A42, A42.8, A42.9, A43, A43.1, A43.9, B35, B35.0, B35.1 – B35.6, B35.8, B35.9, B36, B36.0 – B36.3, B36.8, B36.9, B37, B37.0, B37.2, B37.8, B37.9, B38, B38.3, B38.9, B40, B40.3, B40.9, B42, B42.1, B42.9, B43, B43.0, B43.2, B43.9, B45, B45.2, B45.9, B46.3, B46.5, B47, B47.0, B47.1, B47.9, B48, B48.8 |
| **Tinea capitis** | B35.0 |
| **Other fungal skin disease** | A42, A42.8, A42.9, B35, B35.1 – B35.6, B35.8, B35.9, B36.0 – B36.3, B36.8, B36.9, B37.0, B37.2, B37.8, B37.9, B38.3, B38.9, B40.3, B40.9, B42.1, B42.9, B43, B43.0, B43.2, B43.9, B45, B45.2, B45.9, B46.3, B46.5, B47, B47.0, B47.1, B47.9, B48, B48.8 |
| **Bacterial skin disease** | A20.1, A22, A22.0, A22.9, A26.0, A32.0, A32.9, A36.3, A36.9, A46, A66.0-A66.9, A67.0 – A67.3, A67.9, L00, L05.0, L05.9, L01.0, L01.1, L02.0, L02.1, L02.2, L02.3, L02.4,L02.8L02.9L08.0, L08.1, L08.8,L08.9, L98.0, L88 |
| **Impetigo** | L01, L01.0, L01.1 |
| **Pyoderma** | L02, L02.0-L02.4, L02.8, L02.9, L08, L08.0, L08.1, L08.8, L08.9, L98.0, L88 |
| **Cellulitis** | L03, L03.0-L03.3, L03.8, L03.9 |
| **Abscess and other bacterial skin diseases** | A46, A66.0-A66.9, A67.0 – A67.3, A67.9, L05, L05.0, L05.9 |
| **Skin Malignancy** |  |
| **Melanoma** | C43, C43.0-C43.9, D03, D03.0-D03.3, D03.30, D03.39, D03.4-D03.9, D22, D22.0 - D22.7, D22.9, D48.5 |
| **Non-melanoma skin cancers^†^** | C44.0, C44.1, C44.2, C44.3, C44.4, C44.5, C44.6, C44.7, C44.8, C44.9 |
| **Carcinoma**  **in situ** | D04.0, D04.1, D04.2, D04.3, D04.4, D04.5, D04.6, D04.7, D04.8, D04.9 |
| **Other skin and subcutaneous diseases**  **(Miscellaneous skin conditions)** | A51, A51.0, A51.1, A51.2, A51.3, A51.9, A53, A53.0, A53.9, A57, B68, B68.9, B69, B69.9, B76, B76.0, B76.1, B76.8, B76.9, B78, B78.1, B78.9, B83.1, B55.1, B55.2, B55.9, B85, B87, B88, B89, L10, L10.0 – L10.5, L10.8, L10.9, L11, L11.0, L11.1, L11.8, L11.9, L12, L12.0 – L12.3, L12.8, L12.9, L13, L13.0, L13.1, L13.8, L13.9, L14, L41, L41.0, L41.1, L41.3 – L41.5, L41.8, L41.9, L42, L43.0 – L43.3, L43.8, L43.9, L44, L44.0 – L44.4, L44.8, L44.9, L45, L51, L51.0 – L51.2, L51.8, L51.9, L52, L53, L53.0, L53.3, L53.8, L53.9, L54, L55, L55.0 – L55.2, L55.9, L56, L56.0 – L56.4, L56.8, L56.9, L57, L57.0 – L57.5, L57.8, L57.9, L58, L58.0, L58.1, L58.9, L59, L59.0, L59.8, L59.9, L60, L60.0 – L60.5, L60.8, L60.9, L62, L64, L64.0, L64.8, L64.9, L65, L65.0 – L65.2, L65.8, L65.9, L66, L66.0 – L66.4, L66.8, L66.9, L67, L67.0, L67.1, L67.8, L67.9, L68, L68.0 – L68.3, L68.8, L68.9, L70.1 – L70.5, L70.8, L70.9, L71, L71.1, L71.8, L71.9, L72, L72.1, L72.2, L72.8, L72.9, L73, L73.0 – L73.2, L73.8, L73.9, L74, L74.0 – L74.4, L74.8, L74.9, L75, L75.0 – L75.2, L75.8, L75.9, L80, L81, L81.0 – L81.9, L82, L83, L84, L85, L85.0 – L85.3, L85.8, L85.9, L86, L87, L87.0 – L87.2, L87.8, L87.9, L90, L90.0 – L90.6, L90.8, L90.9, L91, L91.0, L91.8, L91.9, L92, L92.0 – L92.3, L92.8, L92.9, L93, L93.0 – L93.2, L94, L94.0 – L94.6, L94.8, L94.9, L95, L95.0, L95.1, L95.8, L95.9, L97, L98, L98.0 – L98.4,L98.5 – L98.9, L99 |
| **Acne vulgaris** | L70, L70.0 |
| **Psoriasis** | L40, L40.0 – L40.5, L40.8, L40.9 |
| **Alopecia areata** | L63, L63.0 – L63.2, L63.8, L63.9 |
| **Pruritus** | L29, L29.0 – L29.3, L29.8, L29.9 |
| **Urticaria** | L50, L50.0 – L50.6, L50.8, L50.9 |
| **Decubitus ulcer** | L89, L89.0, L89.1, L89.2, L89.3, L89.9 |
| **Scabies** | B86 |

**^†^** Non-melanoma skin cancers included squamous cell carcinoma and basal cell carcinoma

**Supplementary Table 2.** Number of deaths and YLLs for six skin diseases overall and by sex

| **Skin diseases** | **Females** | | **Males** | | **Total** | |
| --- | --- | --- | --- | --- | --- | --- |
|  | **Number of deaths** | **YLL**  **(95% UI)** | **Number of deaths** | **YLL**  **(95% UI)** | **Number of deaths** | **YLL**  **(95% UI)** |
| Cellulitis | 281 | 5,030.74  (4,671.70–5,389.78) | 325 | 5,663.82  (5,336.04–5,991.60) | 606 | 10,694.56  (10,222.44–11,166.68) |
| Abscess and other bacterial skin disease | NA | NA | NA | NA | NA | NA |
| Decubitus ulcer | 147 | 2,141.85  (1,964.77–2,318.94) | 137 | 2,511.87  (2,272.39–2,751.35) | 284 | 4,653.72  (4,316.13–4,991.32) |
| Other skin and subcutaneous disease | 101 | 1,921.82  (1,719.76–2,123.88) | 132 | 2,613.48  (2,329.76–2,897.20) | 233 | 4,535.30  (4,211.51–4,859.09) |
| Non-melanoma skin cancers**^†^** | 12 | 179.62  (127.89–231.35) | 23 | 409.89  (310.63–509.15) | 35 | 589.51  (477.16–701.86) |
| Melanoma | 8 | 120.42  (91.99–148.85) | 16 | 272.28  (219.97–324.59) | 24 | 392.70  (314.05–471.35) |

**Note** 95% uncertainly intervals of YLDs computed using a parametric bootstrap technique

YLLs = Years of life lost

**^†^** Non-melanoma skin cancers included squamous cell carcinoma and basal cell carcinoma

**Supplementary Table 3.** Number of cases and YLDs for 21 skin diseases, overall and by sex

| **Skin diseases** | **Females** | | | **Males** | | | **Total** |
| --- | --- | --- | --- | --- | --- | --- | --- |
|  | **Number of cases** | **Averaged disability weight** | **YLD**  **(95% UI)** | **Number of cases** | **Averaged disability weight** | **YLD**  **(95% UI)** | **YLD**  **(95% UI)** |
| Atopic dermatitis | 591 | 0.264 | 156.02  (142.71– 169.34) | 378 | 0.264 | 99.79  (89.59–110.00) | 255.82  (239.45–272.18) |
| Contact dermatitis | 8,975 | 0.108 | 969.30  (947.81– 990.79) | 5,820 | 0.108 | 628.56  (611.06– 646.06) | 1,597.86  (1,572.32–1,623.40) |
| Seborrheic dermatitis | 360 | 0.014 | 5.04  (4.47–5.61) | 247 | 0.014 | 3.46  (2.94–3.98) | 8.50  (7.95–9.05) |
| Psoriasis | 506 | 0.264 | 133.58  (122.30– 144.87) | 672 | 0.264 | 177.41  (165.00– 189.82) | 310.99  (293.30–328.68) |
| Cellulitis | 7,517 | 0.063 | 473.57  (462.48– 484.67) | 8,124 | 0.063 | 511.81  (500.01– 523.61) | 985.38  (969.43–1001.34) |
| Impetigo | 2,774 | 0.006 | 16.64  (15.98– 17.30) | 2,906 | 0.006 | 17.44  (16.79– 18.08) | 34.08  (33.13–35.03) |
| Abscess and other bacterial skin disease | 11 | 0.006 | 0.07  (0.03–0.10) | 3 | 0.006 | 0.02  (0.00–0.04) | 0.08  (0.04–0.13) |
| Scabies | 98 | 0.027 | 2.65  (2.02–3.27) | 97 | 0.027 | 2.62  (2.02–3.22) | 5.27  (4.57–5.96) |
| Tinea capitis | 214 | 0.006 | 0.95  (0.80–1.10) | 159 | 0.006 | 1.28  (1.07–1.50) | 2.24  (2.01–2.46) |
| Other fungal skin disease | 7,993 | 0.006 | 47.96  (46.95–48.97) | 5,054 | 0.006 | 30.32  (29.60–31.05) | 78.28  (76.75–79.82) |
| Wart | 287 | 0.037 | 10.62  (9.30–11.94) | 209 | 0.037 | 7.73  (6.81–8.65) | 18.35  (16.59–20.11) |
| Molluscum contagiosum | 7 | 0.037 | 0.26  (0.05–0.47) | 5 | 0.037 | 0.19  (0.04–0.33) | 0.44  (0.16–0.73) |
| Acne vulgaris | 63 | 0.124 | 7.81  (5.99–9.64) | 29 | 0.124 | 3.60  (2.40–4.79) | 11.41  (8.70–14.12) |
| Alopecia areata | 123 | 0.039 | 4.80  (3.77–5.82) | 53 | 0.039 | 2.07  (1.50–2.63) | 6.86  (5.94–7.79) |
| Pruritus | 3,632 | 0.011 | 39.95  (38.81–41.10) | 1,612 | 0.011 | 17.73  (16.82–18.65) | 57.68  (56.39–58.97) |
| Urticaria | 8,205 | 0.108 | 886.14  (869.41– 902.87) | 5,175 | 0.108 | 558.90  (543.36– 574.44) | 1,445.04  (1,421.37–1,468.71) |
| Decubitus ulcer | 273 | 0.264 | 72.07  (63.37–80.78) | 304 | 0.264 | 80.26  (72.85–87.66) | 152.33  (139.82–164.84) |
| Other skin and subcutaneous disease | 8,967 | 0.006 | 53.80  (52.41–55.19) | 6,535 | 0.006 | 39.21  (38.22–40.20) | 93.01  (91.68–94.35) |
| Non-melanoma skin cancers**^†^** | 128 | 0.218 | 27.90  (22.58–33.22) | 94 | 0.218 | 20.49  (16.79–24.20) | 48.40  (41.94–54.85) |
| Melanoma | 310 | 0.332 | 102.92  (90.89–114.95) | 125 | 0.332 | 41.50  (34.83–48.17) | 144.42  (129.89–158.95) |
| All skin diseases | NA | NA | 3,013.472  (2,978.44– 3,048.50) | NA | NA | 2,245.416  (2,209.76– 2,281.07) | 5,258.89  (5,206.08–5,311.70) |

**Note** 95% uncertainly intervals of YLDs computed using a parametric bootstrap technique

YLDs = Years lost due to disability

**^†^** Non-melanoma skin cancers; squamous cell carcinoma and basal cell carcinoma
